# Relationship between IL-6 levels and therapeutic adherence in a sample of schizophrenic inpatients

**DOI:** 10.1192/j.eurpsy.2026.10541

**Published:** 2026-07-31

**Authors:** E. Diaz-Mesa, A. Morera-Fumero, A. Aparicio-Dominguez, C. Baez-Marrero, A. M. Hamilton-Lopez, P. Abreu-Gonzalez, M. D. R. Cejas-Mendez, L. Fernandez-Lopez

**Affiliations:** 1 Service of Psychiatry, University Hospital of the Canaries; 2 Deparment of Internal Medicine Dermatology and Psychiatry; 3 Deparment of Physiology, Shool of Medicine, La Laguna, Spain

## Abstract

**Introduction:**

Schizophrenia is a chronic and severe psychiatric disorder characterized by relapses. Non adherence to medication is a major concern, with rates reported between 40% and 50%. Poor adherence is associated with relapse, increased hospitalizations, and a higher risk of suicide (Vega et al., Compr Psychiatry 2021;108:152240). Both direct (drug or metabolite measurement in biological fluids) and indirect methods (tablet counts, electronic monitoring, pharmacy records, etc.) have been used to assess adherence. However, research on biological markers of adherence remains scarce.

**Objectives:**

The aim of this study was to investigate whether serum interleukin-6 (IL-6) levels differ according to antipsychotic treatment adherence.

**Methods:**

The sample included 43 patients who met ICD-10 criteria for paranoid schizophrenia and were in an acute psychotic phase. Blood samples were collected on the first day after admission at 12:00 h and 00:00 h by venipuncture. All patients were receiving antipsychotics due to clinical decompensation. Most patients (N = 15; adherence in the rest unknown) were admitted after treatment discontinuation, while a small subsample presented irregular adherence. Only a minority were strictly adherent (N = 7).

IL-6 was measured using ELISA techniques (Kikugawa et al., Anal Biochem 1992;202:249–55). Patients were treated in the Emergency Department of the University Hospital of the Canary Islands for acute psychotic decompensation and were admitted to the acute psychiatric unit between January 1 and June 1, 2006. Most patients were referred by Mental Health Units, family members, or emergency services; a small percentage (3.7%) presented on their own initiative. Informed consent was obtained either from the patient or, in cases of severe clinical status, from family members.

Comparisons between quantitative variables were performed using Student’s t-test for independent samples.

**Results:**

Serum IL-6 levels at 12:00 h on the day after admission were 0.39 (SD missing), while IL-6 levels at 00:00 h were 0.20 (SD missing).

Next table shows the comparison of serum IL-6 at 12:00 h and 00:00 h between adherent and non-adherent groups.

**Table 1.** Table 1. Long description.

Adherence	N	Mean	Standard Deviation	p
IL-6 12:00 (Yes)	7	4.90	1.84	0.37
IL-6 12:00 (No)	11	6.15	3.26	0.37
IL-6 00:00 (Yes)	7	5.99	3.41	0.55
IL-6 00:00 (No)	14	6.67	1.85	0.55

**Conclusions:**

Serum IL-6 levels at admission did not differentiate between patients who adhered to treatment and those who did not.

**Disclosure of Interest:**

None Declared

## Long descriptions

### Table 1. Long description

The table consists of five columns: Adherence, N, Mean, Standard Deviation, and p.

* Row 1: I L-6 12 00 (Yes), N is 7, Mean is 4.90, Standard Deviation is 1.84, p is 0.37.

* Row 2: I L-6 12 00 (No), N is 11, Mean is 6.15, Standard Deviation is 3.26, p is 0.37.

* Row 3: I L-6 00 00 (Yes), N is 7, Mean is 5.99, Standard Deviation is 3.41, p is 0.55.

* Row 4: I L-6 00 00 (No), N is 14, Mean is 6.67, Standard Deviation is 1.85, p is 0.55.

Navigate back to Table 1..

